# The impact of the COVID-19 pandemic on the career choice of medicine: A cross-sectional study amongst pre-medical students in Pakistan

**DOI:** 10.1016/j.amsu.2022.104219

**Published:** 2022-08-07

**Authors:** Raisa Saleh, Russell Seth Martins, Muhammad Saad, Asad Saulat Fatimi, Gaurav Kumar, Manzar Abbas, Inaara Akbar, Hamzah Jehanzeb, Shamila Ladak, Shamama Kaleem, Sarah Nadeem

**Affiliations:** a Medical College, Aga Khan University Hospital, Karachi, Pakistan; b Society for Promoting Innovation in Medicine (SPIE), Center for Innovation in Medical Education (CIME), Aga Khan University, Karachi, Pakistan; c Nixor College, Karachi, Pakistan; d Medical College, Dow University of Health Sciences, Karachi, 74200, Pakistan; e Section of Endocrinology, Department of Medicine, Aga Khan University, Karachi, 74800, Pakistan

**Keywords:** Medical education, Specialization, Motivations, Deterrents, Pre-medical

## Abstract

**Background:**

The COVID-19 pandemic has significantly affected the lives of healthcare workers due to the frontline nature of their work. Their hard work and sacrifice have forged new perceptions of healthcare workers. These changes may potentially influence students' interest in medicine. This study explores how the COVID-19 pandemic has affected premedical students’ decisions to pursue medicine as a career.

**Methods:**

A cross-sectional study using a self-designed online questionnaire was carried out amongst pre-medical students across Pakistan.

**Results:**

A total of 1695 students from 93 public and private schools filled in the survey. After the onset of the COVID-19 pandemic, significantly more pre-medical students want to pursue medicine (60.7%–62.9%) and less are unsure (20.2%–17%). Students are significantly more likely to be motivated to pursue medicine due to altruistic benefits to society (57% vs. 62.7%) and be deterred by the risk of contracting infections on duty (10%–14.6%). There is a minor but significant increase in the popularity of internal medicine (17.1%–18.9%), public health (4.1% vs. 5.7%), emergency medicine (3.8% vs. 5.7%), pediatrics (3.8% vs. 4.7%), and radiology (2.1% vs. 2.9%). Most pre-medical students felt that doctors routinely undergo physical and emotional turmoil (84%).

**Conclusions:**

Although awareness of hardships faced by medical professionals has increased, motivation to pursue medicine has grown. Through understanding trends in the motivations of students to pursue medicine, medical schools can accommodate the expectations of incoming students and reach out to potential applicants.

## Highlights

• More pre-medical students want to pursue medicine since the onset of the pandemic.
• The major motivation to pursue medicine remains altruistic.
• Since the pandemic, students are more likely to be deterred by occupational risks.
• Some specialties have become more popular choices since the pandemic.
• These include pediatrics, public health, and internal medicine.

## List of abbreviations

COVID-19: Coronavirus Disease 2019
SARS: Severe Acute Respiratory Syndrome
MCAT: Medical College Admission Test
IBM: International Business Machines
SPSS: Statistical Package for Social Sciences
PKR: Pakistani Rupee

## 1 Background

Since the onset of the COVID-19 pandemic, healthcare workers (HCW) have worked long hours under grueling circumstances at great personal risk [[1], [2], [3]], with repercussions on their mental, physical, and emotional wellbeing [[3], [4], [5]]. However, the pandemic has also made the general public increasingly cognizant of the challenges faced by HCWs, with media and government officials highlighting their indispensable role. Many have demonstrated their appreciation for those working in healthcare through collectively celebrating them [6]. However, in many cases, the pandemic has led to individuals distrusting HCWs, with the opinion that they are misrepresenting the severity of the disease or even falsely diagnosing patients with COVID-19 in return for a payout [7,8].

These shifting perceptions of HCWs have affected people from all walks of life. One population that may be particularly impacted are those aspiring to become doctors [9,10]. Around 20% of medical students in the United States of America (US) feel that their choice of specialization has been impacted by the pandemic [11]. With this pandemic bringing the rigors and challenges of the medical profession to the public spotlight, it is uncertain how the motivations of aspiring medical students have been affected. In the US, the number of medical school applications has increased during the pandemic [12]. However, there is less data on the impact of the pandemic in other parts of the world, particularly lower income countries where the motivators and deterrents of a career in medicine vary drastically [10,13,14].

Therefore, this study explores how the COVID-19 pandemic has affected premedical students’ decisions to pursue medicine as a career in Pakistan. The overburdened nature of the healthcare system in Pakistan and the shortage of HCWs makes it particularly important to explore the impact of the pandemic on motivators and deterrents influencing students considering a career in medicine.

## 2 Materials and methods

A cross-sectional survey was conducted amongst pre-medical students in Pakistan. This study received ethical approval from the Ethical Review Committee at the Aga Khan University, Pakistan. This study was also registered on ClinicalTrials.gov (NCT05379465) [15]. All work has been reported in line with the STROCSS criteria [16].

### 2.1 Pre-medical students in Pakistan

Our target population consisted of pre-medical students in Pakistan between the ages of 14–20 years. In Pakistan pre-medical students are high school students, as medicine is a direct-entry undergraduate program after high school. The two most widely followed educational boards in Pakistan that offer pre-medical high school education are the National Board of Intermediate and Secondary Education (BISE) in Pakistan (levels include Matriculation 1–2 and Intermediate 1–2), and the Cambridge Assessment International Education (CAIE) board (levels include O-Levels Grades 9–11 and A-Levels 1–2). Other less commonly followed boards include the Aga Khan University Examination Board (AKU-EB) and the International Baccalaureate (IB). High-school students in the pre-medical stream study, at a minimum, the subjects of biology, physics and chemistry in high school, as these are the pre-requisites to applying to medical school. Students are required to pass a national Medical College Admission Test (MCAT) to apply to public and private medical colleges. It is important to note, however, that though only high-school students within the pre-medical stream are eligible to apply to medical school, these students are also eligible to apply to other non-medical disciplines of higher education.

### 2.2 Sample size and population

The total population size of pre-medical students in Pakistan in any given year ranges from 120,000–150,000 [17,18]. To err on the side of a larger minimum sample size, we used a population size of 150,000 for our calculations. The minimum sample size calculated for this study was 384, assuming a precision level of 5% and an anticipated frequency of positive perceptions of medicine of 50%, with a confidence interval of 95%. OpenEpi software was used to calculate the required sample size [19].

### 2.3 Data collection tool and coding

In the absence of any existing validated surveys suitable for this study, the questionnaire used was self-designed by the research team. The questionnaire was in English, as English is the primary language of instruction in medical schools and high school across Pakistan. This was first pre-tested on a population of 25 pre-medical students belonging to 5 different educational institutions in Pakistan. One question was removed during pretesting due to repetition.

The questionnaire was disseminated as an anonymous Google Form survey, which was preceded by a consent form. Since a large subset of our target population was <18 years, the participants were asked to seek their parents’ consent before filling the form.

The final questionnaire consisted of the following three mandatory sections:

1. **Demographics and Educational Institution Details:** Students' age, gender, socio-economic background (area of residence and ethnicity), education system, year of study, and academic stream were collected. In addition, students were asked if they had any first-degree relatives who are doctors. Despite the inclusion of several socio-demographic identifiers, the nationwide dissemination of the survey ensured anonymity of responses.
2. **Career Choices before vs. after the onset of COVID-19 Pandemic:** Students were asked whether they wanted to pursue medicine prior to vs. after the onset of the pandemic, to what degree they were motivated to pursue medicine (rated on a 5-point Likert scale) and which specialties of medicine, if any, they were interested in. They were then asked to rate on a 3-point Likert scale the importance of various motivators and deterrents on their motivation to pursue medicine, before and after the onset of the pandemic. If students were no longer considering medicine after the onset of the pandemic, they were asked which career path they were now considering.
3. **Perceptions of Doctors:** Students were asked to indicate agreement (on a 5-point Likert Scale) with statements about doctors' lifestyles and the impact of their work on their social, physical, and emotional wellbeing. Negatively phrased statements were coded oppositely for analysis.

### 2.4 Sampling technique

Given that there was no official platform through which to disseminate the survey in a systematic manner to schools across the country, we were compelled to make the most of a convenience sampling and snowball sampling approach. It was disseminated online via social media platforms as well as through email. The questionnaire was shared on WhatsApp and Facebook groups of various educational institutions in Pakistan as well as on several nationwide MCAT study groups.

Duplicate responses were eliminated by screening for responses with the following identical demographics: age, gender, ethnicity, administrative region/province of residence, board of education, field of education, most recent level of education, school's name, and monthly family income.

### 2.5 Statistical analysis

Statistical analyses were run using International Business Machines (IBM) Statistical Package for Social Sciences (SPSS) version 21. Normally distributed continuous data was reported as mean ± standard deviation, whereas categorical data was reported as gross numbers and percentages (n; %). Unpaired categorical data was compared using Chi-squared tests. Paired categorical data was compared using Mc Nemar's test. A p-value < 0.05 was considered as significant for all analyses.

## 3 Results

### 3.1 Demographics (Table 1)

A total of 1695 students from 93 schools across Pakistan filled the survey, exceeding our minimum required sample size. Nearly three quarters of respondents identified as female (71.9%). The largest number of respondents (46.4%) were from Punjab, and the most common ethnicity was Punjabi (49.3%). The greatest percentage of respondents (46.4%) had a family income of PKR (Pakistani Rupee) 100,000 to 500,000. Most respondents (78.8%) were enrolled in the Cambridge O/A Levels board, and the most common level of education was AS levels or equivalent. Most respondents (72.8%) did not have a doctor in the immediate family. The demographic data of the participants is shown in Table 1.

**Table 1:** Demographic characteristics.

Variable	n (%)/Mean ± SD
**Age (years)**	17.3 ± 1.17
**Gender**	
**Male**	452 (26.7)
**Female**	1219 (71.9)
**Prefer Not to Say**	24 (1.4)
**Administrative Regions**	
**Punjab**	787 (46.4)
**Sindh**	659 (38.9)
**Islamabad Capital Territory**	215 (12.7)
**Balochistan**	18 (1.1)
**Azad Jammu and Kashmir/Gilgit Baltistan**	3 (0.2)
**Ethnicity**	
**Punjabi**	836 (49.3)
**Sindhi**	190 (11.2)
**Muhajir**	160 (9.4)
**Pashtun**	101 (6.0)
**Baloch**	16 (0.9)
**Mixed**	124 (7.3)
**Other**	99 (5.8)
**Prefer Not to Say**	169 (10.0)
**Monthly Family Income**	
**< PKR 50,000**	146 (8.6)
**PKR 50,000**–**100,000**	334 (19.7)
**PKR 100,000**–**500,000**	787 (46.4)
**> PKR 500,000**	428 (25.3)
**Board of Education**	
**Cambridge O-/A-Levels**	1336 (78.8)
**Provincial Matriculation/Intermediate**	233 (13.7)
**Federal Board of Intermediate and Secondary Education**	15 (0.9)
**Aga Khan University Educational Board**	103 (6.1)
**Others**	8 (0.5)
**Most Recent Level of Education**	
**O-Levels Grade 9**	91 (5.4)
**O-Levels Grade 10/Equivalent**	116 (6.8)
**O-Levels Grade 11/Equivalent**	470 (27.7)
**AS Levels/Equivalent**	593 (35.0)
**A2 Levels/Equivalent**	425 (25.1)
**Doctor in the Family**	
**Yes**	461 (27.2)
**No**	1234 (72.8)
**If Yes, Who**	**N = 461**
**Father**	208 (45.1)
**Mother**	212 (46.0)
**Sister**	144 (31.2)
**Brother**	44 (9.5)

**Table 2:** Effect of the COVID-19 Pandemic on choice of medical career and specialty of choice.

Variable	Before Pandemic n (%)	After Pandemic n (%)	P-Value
**Did/Do you want to pursue medicine as a career?**			
**Yes**	1029 (60.7)	1066 (62.9)	**0.003**
**No**	324 (19.1)	341 (20.1)	
**Unsure**	342 (20.2)	288 (17.0)	
**I am/was motivated to become a doctor.**			
**Strongly Agree/Agree**	954 (56.3)	1042 (61.5)	**< 0.001**
**Neither Agree nor Disagree**	342 (20.2)	243 (14.3)	
**Strongly Disagree/Disagree**	399 (23.5)	410 (24.2)	
**Specialty of Choice**			
**Surgery**	415 (24.5)	400 (23.6)	0.235
**Internal Medicine or Subspecialties**	289 (17.1)	320 (18.9)	**0.012**
**Neurology**	264 (15.6)	286 (16.9)	0.059
**Dermatology**	169 (10.0)	144 (8.5)	**0.007**
**Psychiatry**	149 (8.8)	141 (8.3)	0.403
**Family Medicine**	81 (4.8)	87 (5.1)	0.544
**Public Health**	69 (4.1)	96 (5.7)	**0.004**
**Emergency Medicine**	64 (3.8)	97 (5.7)	**< 0.001**
**Pediatrics**	64 (3.8)	80 (4.7)	**0.038**
**Genetics**	57 (3.4)	70 (4.1)	0.093
**Anesthesiology**	47 (2.8)	58 (3.4)	0.127
**Radiology**	35 (2.1)	50 (2.9)	**0.012**
**Pathology**	32 (1.9)	42 (2.5)	0.165
**Ophthalmology**	25 (1.5)	26 (1.5)	>0.999
**Undecided**	447 (26.4)	482 (28.4)	**0.025**
**Not Interested in Medicine**	296 (17.5)	299 (17.6)	0.876

**Table 3:** Effect of the COVID-19 Pandemic on motivating factors to pursue medicine.

Motivating Factors^a^	Before Pandemic n (%)	After Pandemic n (%)	P-Value
**Personal Desire to Help Others**	1091 (64.4)	1098 (64.8)	0.735
**Medicine is a Career that Benefits Society at Large**	966 (57.0)	1062 (62.7)	**< 0.001**
**Interest in the Science of Medicine**	893 (52.7)	844 (49.8)	**0.005**
**Intellectual Satisfaction**	840 (49.6)	827 (48.8)	0.480
**Future Job Prospects**	771 (45.5)	718 (42.4)	**0.006**
**Research Opportunities**	737 (43.5)	744 (43.9)	0.716
**Like Working with People**	724 (42.7)	672 (39.6)	**0.001**
**Job Security**	724 (42.7)	661 (39.0)	**< 0.001**
**Personality is Suited to Medicine**	671 (39.6)	663 (39.1)	0.629
**Prestige of Becoming a Doctor**	653 (38.5)	674 (39.8)	0.241
**Opportunities to Work/Travel Internationally**	617 (36.4)	621 (36.6)	0.849
**Medicine can be a High-Income Career**	606 (35.8)	626 (36.9)	0.261
**Doctor in the Family served as a Role Model**	398 (23.5)	349 (20.6)	**0.001**
**Loss of Loved One to an Illness**	346 (20.4)	349 (20.6)	0.883
**Opportunity to use Cutting-Edge Medical Technology**	324 (19.1)	385 (22.7)	**< 0.001**
**Family Pressures**	241 (14.2)	212 (12.5)	**0.028**
**Too Late to Switch Career Path**	212 (12.5)	224 (13.2)	0.420
**Wanted to Continue a Family Tradition**	182 (10.7)	171 (10.1)	0.315

a Only responses marked “Significant Impact” shown; “No/Insignificant Impact” not shown.

**Table 4:** Effect of the COVID-19 Pandemic on deterrents to pursue medicine.

Deterring Factors	Before Pandemic n (%)	After Pandemic n (%)	P-Value
**Long Duration of Education and Training**	492 (29.0)	441 (26.0)	**0.003**
**Stressful Working Conditions**	385 (22.7)	399 (23.5)	0.424
**Education and Training are Too Challenging**	356 (21.0)	360 (21.2)	0.845
**Inability to Apply to Medical College due to Grades**	330 (19.5)	350 (20.6)	0.128
**Inability to Finance Medical Education**	287 (16.9)	329 (19.4)	**0.001**
**Concerns Regarding Job Opportunities**	262 (15.5)	260 (15.3)	0.946
**Not Interested in the Field**	217 (12.8)	195 (11.5)	0.074
**Risk of Contracting Serious Illnesses on Duty**	169 (10.0)	248 (14.6)	**< 0.001**
**Public Distrust of Doctors**	158 (9.3)	171 (10.1)	0.353
**Risk of Violence from Patients/Attendants**	124 (7.3)	172 (10.1)	**< 0.001**
**Personal Distrust of Doctors**	115 (6.8)	142 (8.4)	**0.029**
**No Intention of Pursuing Further Education**	108 (6.4)	118 (7.0)	0.395
**Family wants me to Pursue Other Career**	91 (5.4)	119 (7.0)	**0.003**

*Only responses marked “Significant Impact” shown; “No/Insignificant Impact” not shown.

### 3.2 Effect of the COVID-19 pandemic on choice of medical career (Table 2)

Prior to the pandemic, 60.7% of the study's participants wanted to pursue medicine, with 56.3% agreeing/strongly agreeing that they were motivated to become doctors. The most popular subspecialty within medicine was surgery, with 24.5% choosing it as their specialty of choice, although the largest percentage of respondents (26.4%) were undecided about their specialty.

After the pandemic, 62.9% wanted to pursue medicine, demonstrating a significant increase in the percentage of students wanting to pursue medicine (p = 0.03). Moreover, 61.5% were strongly motivated to become a doctor, showing a significant increase (p value < 0.01) since the start of the pandemic.

Surgery remained the most popular specialty, even after the onset of the pandemic, with 23.6% wanting to pursue surgery. A significantly larger percentage of respondents were undecided about their specialty as compared to before the pandemic. Five subspecialties showed a statistically significant increase in popularity after the onset of the pandemic: internal medicine or specialties, public health, emergency medicine, pediatrics, and radiology. On the other hand, only dermatology showed a significant decrease in popularity.

Of the students not intending to pursue medicine after the onset of the pandemic (n = 939), the greatest percentage (35.4%) reported other health sciences/pure sciences as their desired career path, while the rest favored engineering/computer science (24.9%), business/accountancy (13.3%), law (12.2%), or other careers (11.1%). The remaining 3.1% (n = 29) students did not intend to pursue further education.

### 3.3 Effect of the COVID-19 pandemic on motivating factors (Table 3)

Of the factors motivating students to pursue medicine prior to the pandemic, the commonest were a personal desire to help others (64.4%), medicine being a career that benefits society at large (57%) and an interest in the science of medicine (52.7%). Even after the pandemic, a personal desire to help others (64.8%), medicine being a career that benefits society at large (62.7%) and an interest in the science of medicine (49.8%) remained the three most cited motivators.

Two motivating factors showed a statistically significant increase in popularity compared to the beginning of the pandemic: medicine being a career that benefits society at large, and opportunities to use cutting-edge medical technology. In addition, six motivating factors showed a significant decrease: family pressures, an interest in the science of medicine, enjoying working with people, future job prospects, a doctor in the family served as a role model, and job security.

### 3.4 Effect of the COVID-19 pandemic on deterring factors (Table 4)

Amongst the deterring factors, the long duration of education and training (29.0%), stressful working conditions (22.7%) and the challenging education and training (21.0%) were most often cited as deterrents to pursuing a career in medicine prior to the pandemic. The three most cited deterring factors remained the same after the start of the pandemic.

Five deterring factors showed a significant increase since the start of the pandemic: inability to finance medical education, family wanting one to pursue other career, a personal distrust of doctors, the risk of violence from patients/attendants, and risk of contracting serious illness on duty. Only one deterring factor showed a decrease: the long duration of education and training.

### 3.5 Perceptions of doctors

**Fig. 1:**
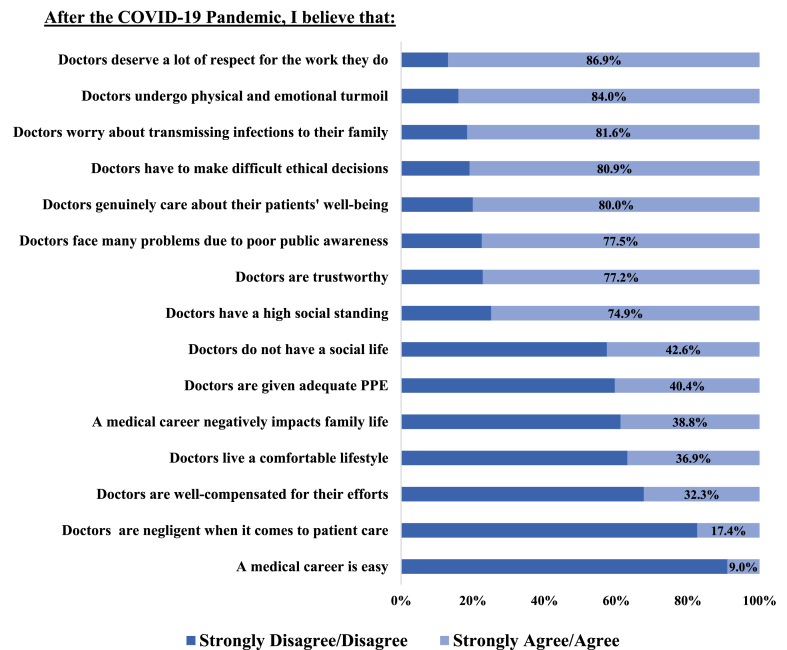
Perceptions of a Medical Career after the COVID-19 Pandemic *All “unsure” responses were eliminated for the above figure.

We collapsed the 5-point Likert Scales to a 3-point scale (Strongly Disagree/Disagree, Unsure, Strongly Agree/Agree), and participants responding Unsure to any statement were excluded. The results for this section are shown in Fig. 1.

After the onset of the pandemic, most participants had positive perceptions of doctors but were also aware of risks associated with working in the field of medicine. There were high levels of agreement with the statements “doctors deserve a lot of respect for the work they do” (86.9%), “doctors undergo physical and emotional turmoil” (84.0%) and “doctors worry about transmitting infections to their family” (81.6%). Conversely, most participants did not agree with the statements “a medical career is easy” (9.0%), “doctors are negligent when it comes to patient care” (17.4%) and “doctors are well-compensated for their efforts” (32.3%).

### 3.6 Discussion

To our knowledge, ours is the first study that assesses the impact of the COVID-19 pandemic on the career choice of medicine amongst pre-medical students. Our results revealed that even though students are now aware of the adverse physical and mental effects of the COVID-19 pandemic on frontline health workers, the overall perceptions regarding the medical field have improved.

We observed a small but significant increase in the number of pre-medical students choosing to pursue medicine after the onset of the COVID-19 pandemic, with a concurrent decrease in the number of students who were unsure. This was also reflected by an increase in motivation to pursue medicine. These increases are especially interesting since most students displayed an awareness of the hardships faced by HCWs during the pandemic. It is important to note, however, that because participants of our study were asked to recall their desire to pursue medicine prior to the pandemic several months after it began, there may be an element of recall bias when looking at the pre-pandemic data.

Most students agreed that doctors undergo physical and emotional turmoil (84%) and often have to make difficult ethical decisions (80.9%) as part of their job. Moreover, the majority disagreed that a career in medicine was easy (91%), that doctors are well-compensated for their efforts (67.7%), and that doctors are given adequate personal protective equipment (59.6%). To add to this, students also more commonly reported being deterred because their family wanting one to pursue some other career. Notably, despite the pandemic portraying the more grueling aspects of the field, more students are motivated towards pursuing medicine. An increase in altruism motivating pre-medical students after the COVID-19 pandemic can been seen by how a significantly higher percentage of respondents were motivated because medicine benefits society at large. Additionally, more students reported wanting to pursue medicine because it provides the opportunity to use cutting-edge medical technology, likely inspired by the global use of medical technology in COVID-19 diagnosis, management, and vaccine development. These results are particularly relevant for lower-middle-income countries like Pakistan, where health systems already suffer from a perennial shortage of HCWs and physicians [20].

Our results show significant increases in the popularity of certain specialties amongst pre-medical students. As compared to before the COVID-19 pandemic, more students reported considering specializing in internal medicine or its subspecialties, public health, emergency medicine, pediatrics, and radiology. These results may be explained by a variety of reasons. Since HCWs in infectious disease (one of the subspecialties of internal medicine) and public health have been at the forefront of media coverage during the COVID-19 pandemic, the increased exposure to these specialties is likely to have piqued the interest of aspiring doctors. Similarly, emergency medicine doctors, who have been at the frontline of the COVID-19 response, and pediatricians, who have had to combat the unique manifestations of COVID-19 in children [21], have also been in the public eye. Interestingly, the increase in the interest towards a career in radiology may be explained by the lesser amount of patient interaction that the specialty entails, which translates to less exposure to transmissible diseases and other hazards such as violence against physicians. This is evidenced by how 81.6% of respondents in our study believed that doctors have to worry about passing on infections to their family, and how a significantly greater percentage of respondents were likely to be deterred by the risk of contracting infections and the risk of violence from patients or attendants.

Other deterrents more commonly reported after the pandemic were an inability to finance a medical education and a personal distrust in doctors. The former may be due to the financial losses suffered by families due to the pandemic, whereas the latter may be due to propaganda and conspiracy theories that have surfaced during the COVID-19 pandemic in Pakistan. These include notions that doctors are misrepresenting the severity of the disease or even falsely diagnosing patients with COVID-19 in return for a payout [7,8].

External motivators less such commonly reported to be reasons for pursuing medicine after the COVID-19 pandemic include future job prospects, job security, having a doctor in the family as a role model, and family pressures. These results are interesting, as they suggest that the revelation of the realities of a medical career in the pandemic may serve to better inform pre-medical students and negate the effects of external influences on their decision to pursue medicine.

Limitations of this study include it being limited to only the Pakistani population thus providing data limited to a single country. Within Pakistan, however, our results are extremely generalizable, as respondents belonged to varying socio-economic and ethnic backgrounds, and hailed from 93 schools across the country. A key strength of our study is its large sample size, which we were able to achieve by leveraging the outreach of social media in an exhaustive data collection process. We were unable to calculate a response rate due to convenience sampling over the internet. Nevertheless, our article is the first of its kind and provides an important perspective on how the COVID-19 pandemic has changed how potential future doctors view the practice of medicine and its subspecialties.

## 4 Conclusion

The COVID-19 pandemic has resulted in shifting perspectives of prospective medical school applicants about the field of medicine. Although awareness of hardships faced by medical professionals has increased, the number of students motivated to pursue medicine has also grown. The motivators to pursue medicine remain majorly altruistic. However, students were more concerned about the risks of contracting illnesses at work and of violence from patients. Through understanding these trends in the motivations of students to pursue medicine, educational institutions can prepare to accommodate the expectations of incoming students as well as to reach out to potential applicants.

## Ethics approval and consent to participate

We received ERC approval (ERC Number: 2020-5368-14553), which was necessary due to the study involving the collection of data from individuals.

**Table:** 

PUSHED

## Funding

None.

## Competing interests

The authors declare that they have no competing interests.

## Author contribution

Raisa Saleh (RS) and Russell Seth Martins (RSM) conceptualized and supervised the investigation, and had major contributions in devising the methodology, analyzing the data, and writing and editing the manuscript. Manzar Abbas (MA) was a major contributor in analyzing the data. Asad Saulat Fatimi (ASF), Shamila Ladak (SL), Hamzah Jehanzeb (HJ), Gaurav Kumar (GK), Shamama Kaleem (SK), Muhammad Saad (MS), Manzar Abbas (MA), and Inaara Akbar (IA) collected the data by virtually administering the survey and were major contributors in writing and editing the manuscript. Sarah Nadeem (SN) critically reviewed and suggested changes in the manuscript. All authors reviewed the results and approved the final manuscript.

## Consent for publication

We obtained written informed consent for publication from all participants.

## Data availability

The datasets used and/or analyzed during the current study are available from the corresponding author on reasonable request.

## Protocol

The protocol is available from the authors upon reasonable request.

## Provenance and peer review

Not commissioned, externally peer reviewed.

## Guarantor

The Guarantor is the one or more people who accept full responsibility for the work and/or the conduct of the study, had access to the data, and controlled the decision to publish.

Sarah Nadeem (MD, FACE).
